# Dominant role of the α-chain in rejection of tumor cells bearing a specific alloantigen in TCRα transgenic mice and in *in vitro* experiments

**DOI:** 10.18632/oncotarget.27093

**Published:** 2019-08-06

**Authors:** Maria Zamkova, Anastasiya Kalinina, Yuliya Silaeva, Nadezhda Persiyantseva, Alexandra Bruter, Alexey Deikin, Ludmila Khromykh, Dmitry Kazansky

**Affiliations:** ^1^ “N. N. Blokhin National Medical Research Centre of Oncology” of the Health Ministry of Russia, Moscow, Russia; ^2^ Russian Academy of Sciences, Engelhardt Institute of Molecular Biology, Moscow, Russia; ^3^ Institute of Gene Biology, Russian Academy of Sciences, Moscow, Russia

**Keywords:** transgenic mice, TCRα, tumor, alloantigen

## Abstract

Both TCRα and TCRβ types of T-cell receptors contribute to antigen recognition. However, some TCRs have chain centricity, which means that either the α-chain or the β-chain dictates the peptide–MHC complex specificity. Most earlier reports investigated the role of well-studied β-chains in antigen recognition by TCRαβ. In a previous study, we identified TCRs specific to the H-2K^b^ molecule. In the present work, we generated transgenic mice carrying the α-chain of this TCR. We found that these transgenic mice rejected EL-4 tumor cells bearing alloantigen H-2K^b^ more effectively than wild-type mice and similarly to mice with established specific memory T cells. Moreover, we found that T cells transduced with this TCRα can inhibit EL-4 cell growth *in vitro* and *in vivo*. We also found that transgenic mice recruit fewer CD8 T cells into the peritoneal cavity at the peak of the immune response and had a significantly higher number of central memory CD8 T cells in the spleen of intact transgenic mice compared to intact wild-type control. These results indicate the ability of a single transgenic α-chain of the H-2K^b^-specific TCR to determine specific recognition of the H-2K^b^ molecule by a repertoire of T lymphocytes and to rapidly reject H-2K^b^-bearing lymphoma cells.

## INTRODUCTION

Each T cell has one or less often two types of T-cell receptors (TCRs) on its surface. The TCR is composed of α- and β-chains. As allelic exclusion of the β-chain occurs, only one β-chain can be rearranged in a single T cell. On the contrary, due to the lack of α-chain allelic exclusion, some mature T cells can express two different TCR α-chains [1]. Both TCRα and TCRβ contain constant (Cα and Cβ, respectively), variable (Vα and Vβ, respectively), joining (Jα and Jβ, respectively) and, in the case of TCRβ, diversity (D) regions. Three extremely variable regions called complementarity-determining regions (CDRs), which are present in both α- and β-chains, are responsible for the TCR specificity. Two of them (CDR1 and CDR2) are encoded by V gene segments, and CDR3 is determined by V(D)/J recombination together with insertion or deletion of random nucleotides. CDR3 makes a major contribution to the recognition of a peptide–MHC complex (pMHC) by a TCR. It was shown that CDR1 and CDR2 interact with a MHC molecule, and the highly variable CDR3 contacts with the unique peptide component of a pMHC [2]. We should mention that studies describing TCR chain recombination were done using murine models. However, the structure of TCR loci is similar in mice and humans; a number of studies describe the level of sequence similarity of TCR repertoires between mouse and human [3, 4].

Adoptive transfer of genetically modified T cells is one of the strategies for killing cancer cells [5]. Engineering of T cells can be implemented by identification of tumor-specific TCRαβ followed by its introduction into autologous T cells [6–8]. Some studies and clinical trials have confirmed the efficacy of such modified T cells for treating cancer [9, 10]. An alternative approach is the construction of CAR (chimeric antigen receptor) T cells that target surface antigens directly [11]. The best targets for CAR and tumor-specific TCRαβ are tumor-specific antigens (TSA), because they are not expressed in normal cells. But as these antigens are highly heterogeneous even among patients with the same type of tumor, it is rather difficult and expensive to create CAR or tumor-specific TCRαβ T cells for most patients [12]. Other targets of modified T cells are tumor-associated antigens (TAAs). But as this type of antigens are also expressed in normal cells, CAR or tumor-specific TCRαβ T cells can cause potentially serious off-tumor toxicities [13, 14]. CAR T cells are mostly effective in treatment of CD19-positive hematologic malignancies [15], whereas TCRαβ T cells target all cellular proteins. Identification of specific TCRαβs is a time-consuming and expensive procedure and includes sequencing of α- and β-chains with their subsequent appropriate pairing [16]. Indeed, identification of only one TCR chain that contributed most to antigen recognition would save time and lower the cost of tumor-specific TCRαβ T-cell therapy.

Although both TCRα and TCRβ contribute to recognition of an antigen, vast experimental data suggest that some TCRs display chain centricity, i.e. either α- or β-chain can dictate the pMHC specificity. Several findings support the idea that TCRα can play the major role in antigen recognition [17, 18]. Yokosuka et al. demonstrated that a TCRα specific to HIVgp160 peptide (RT-1) plays the predominant role in the antigen recognition, and that one third of TCRβ randomly picked from naive T cells of mice can reconstitute the antigen-reactive TCR containing RT-1 TCRα [19]. Nakatsugawa et al. generated a thymically unselected TCR repertoire specific to A2/MART1 composed of a single TCRα paired with various β chains and showed that TCRα determines antigen specificity whereas TCRβ is responsible for the avidity without compromising the specificity. Moreover, Stadinski et al. demonstrated that TCRα can influence the interaction of TCRβ with a pMHC, changing overall TCR specificity [20]. Another group of studies reported that β-chain defines the antigen recognition by a TCR [17]. Zhao et. al. showed that public, but not private, TCRβ specific to the MOG_35-55_ supported antigen recognition by TCR with various α-chains [21]. Ochi et. al. also demonstrated that β-chain of a TCR specific to A24/WT1235 has the dominant role dictating pMHC specificity [18].

Earlier, we identified TCR α- and β-chains that originated from a memory T-cell hybridoma 1D1 specific to the H-2K^b^ molecule [22]. The aim of our subsequent studies was to assess the role of each TCR α- and β-chain in antigen recognition, including evaluation of the ability of single chain transgenic (Tg) mice to eliminate tumor cells harboring the specific antigen. Tg mice (1D1β) expressing a single TCRβ on the genetic background of B10.D2(R101) mice were obtained and characterized as a potential model for studying immunological surveillance [23, 24]. These mice did not reject EL-4 allogenic tumor cells expressing the H-2K^b^ molecule, in contrast to wild-type (WT) (B10.D2(R101)) mice. Moreover, we observed a loss of the H-2K^b^ molecule by EL-4 cells, so they were able to escape from immune response [24]. In the present study, we focused on the TCRα chain that originated from the same memory T-cell hybridoma 1D1 and characterized the role of a single α-chain in specific antigen recognition both *in vitro* and *in vivo*. We created transgenic mice (1D1α) expressing a single TCRα from a memory T-hybridoma specific to the H-2K^b^ molecule on genetic background B10.D2(R101). In contrast to 1D1β mice, 1D1α mice were able to completely eliminate EL-4 tumor cells within 3-6 days from the peritoneal cavity, while WT mice rejected the allogenic tumor in 12 days. This indicates that perhaps the presence of only one chain of an antigen-specific TCR on the surface of a T lymphocyte is sufficient for the lymphocyte to recognize and eliminate cells harboring the target antigen.

## RESULTS

Amino acid analysis revealed that Tg T-cell receptor α-chain 1D1 corresponds to the Vα 11.3 allele of the Vα11 protein family [25]. Its nucleotide and amino acid sequences can be found in GenBank: DQ983579.1. The process of obtaining T-cell hybridoma 1D1 was described earlier [22].

### Elimination of EL-4 cells by 1D1α-transduced T cells *in vitro*

Initially, we assessed the ability of T cells transduced with α-chain 1D1 originally obtained from TCR specific to the alloantigen H-2K^b^ to influence EL-4 cell growth *in vitro*. A retroviral vector containing the sequence of α-chain 1D1 under the PGK promoter was constructed, and T cells expressing this α-chain were obtained by vector transduction into activated mouse T cells. Along with such T cells, we also obtained T cells expressing green fluorescent protein (GFP) in the same experiment. As GFP transcript length is similar to the length of α-chain and it was cloned into the same vector, we assumed that the efficiency of GFP transduction is the same as the efficiency of α-chain transduction. Because there are no commercially available antibodies to detect our α-chain (Vα11.3), we evaluated the efficiency of transduction by measuring the percentage of GFP-transduced T cells by flow cytometry. We also performed PCR to identify the presence of α-chain mRNA in transduced T cells (Supplementary Figure 1A). To detect the expression of α-chain in the T cells directly, we also constructed a retroviral vector containing α-chain fused to GFP.

**Figure 1:**
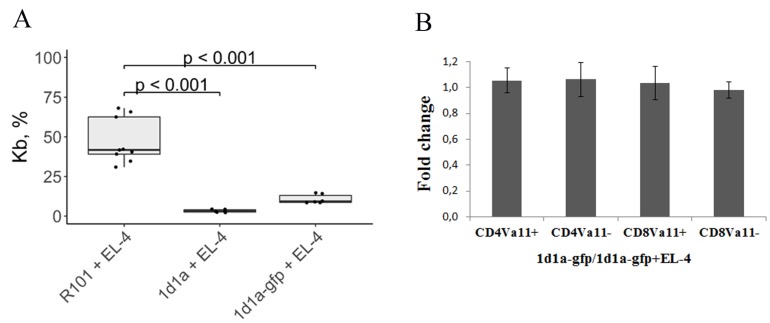
EL-4 cells were cultured with activated lymphocytes transduced with 1D1α and 1D1α-gfp constructions. Analysis of the number of EL-4 cells (**A**) and splenic T-cell phenotype (**B**) were performed 24h after co-culturing. (A) The percentages of Kb positive cells were determined by flow cytometry in three groups of mixed cultures *in vitro* – control (R101 + EL-4) and two experimental (1D1α + EL-4 and 1D1α-gfp + EL-4). (B) The bar graph represents the ratio of CD4Vα11+, CD4Vα11–, CD8Vα11+, and CD8Vα11– in the culture of T cells expressing 1D1α-gfp without EL-4 relative to the culture of T cells expressing 1D1α-gfp along with EL-4. We define 1D1α-gfp positive cells as Vα11+ because GFP matches the cells expressing α-chain 1D1– a member of the Vα11 protein family. The data represent the mean ± sd (*n =* 4–6).

We observed that T cells expressing α-chain with and without fusion could eliminate EL-4 cells from *in vitro* cultures (Figure 1A). Notice that GFP fusion did not interfere with α-chain functionality. Labeling α-chain with GFP allowed us to analyze the phenotype of Vα11 positive and negative T cells cultured alone and together with EL-4 cells. It was mentioned above that T-cell-receptor α-chain 1D1 is a member of Vα11 protein family. We observed no changes in the number of CD4Vα11+, CD4Vα11–, CD8Vα11+, and CD8Vα11 cells in the culture of T cells mixed with EL-4 cells in relation to the culture of T cells alone (Figure 1B). So, we confirmed the ability of T cells expressing a specific single α-chain paired with random endogenously expressed β-chain to eliminate EL-4 cells *in vitro.* Next we decided to evaluate the efficiency of elimination of EL-4 cells *in vivo*. So, we generated transgenic mice carrying the 1D1α chain in their genome.

The cDNA encoding the α-chain of the TCR was cloned into the pTα cassette (a kind gift of Dian Mathis (Institut de Génétique de Biologie Moléculaire et Cellulaire, Strasbourg, France)) [26]. Primary transgenic 1D1α mice were obtained on the genetic background of F1 hybrids (CBA x C57BL/6) as described earlier [23]. To establish the transgenic line, 1D1α primary transgenic mice were backcrossed with B10.D2(R101) mice for 6-7 generations.

### Characterization of transgenic 1D1α mice

**Figure 2:**
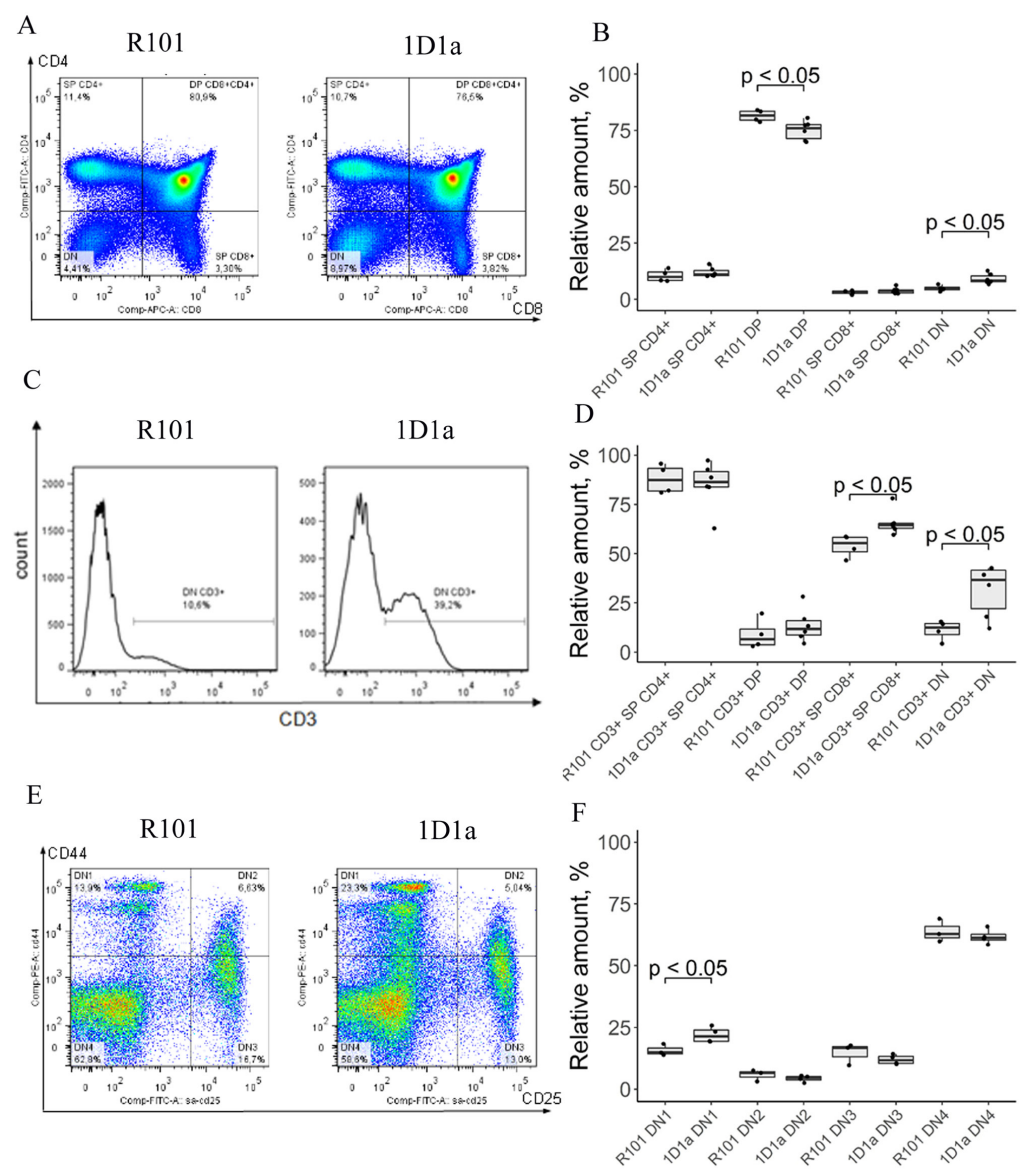
Flow-cytometric analysis of lymphocyte subpopulations in thymus of WT and Tg 1D1α mice. (**A**) Dot plots show expression of CD8 vs CD4 on thymocytes of WT (*left*) and Tg (*right*) mice. (**B**) DN, CD4 SP, and CD8 SP and DP subpopulations in thymus of R101 and 1D1α mice. (**C**) The histogram visualizes the expression of the CD3 marker on DN thymocytes. (**D**) The percentages of CD3+ DN, CD3+ CD4 SP, CD3+ CD8 SP, and CD3+ DP thymocytes are shown. (**E**) Co-expression of CD44 and CD25 on DN-gated thymocytes. (**F**) The box plot shows the distribution of thymocytes over different stages of DN development defined by CD44 and CD25 surface expression. *SP* – single positive, *DN* – double negative, *DP* – double positive. (A), (C), (E) Data from one representative staining are shown.

To evaluate the influence of single transgenic α-chain expression on the development of lymphocytes in the thymus, we analyzed subpopulations of thymocytes in WT and Tg mice. As shown in Figure 2A, 2B, the number of CD4+ single positive (SP) and CD8+ single positive (SP) cells was comparable between WT and Tg mice, but we observed 1.07-fold decrease and 1.9-fold increase in the number of CD8+CD4+ double positive (DP) and CD8–CD4– double negative (DN) cells, respectively, in the thymus of the Tg mice. We also showed that the level of CD3 expression on DN thymocytes and SP CD8 cells of 1D1α mice was 2.8-fold and 1.2-fold higher than on WT thymocytes, respectively (Figure 2C, 2D). Notice that CD3 expression on other thymic subpopulations (i.e. SP CD4 and DP) was similar in WT and Tg mice.

To assess the influence of transgene α-chain expression on early stages of T cell differentiation, we estimated the distribution of CD8–CD4– thymocytes over stages of DN cell development. DN thymocytes are subdivided into DN1, DN2, DN3, and DN4 stages depending on the expression of CD44 and CD25 [27]. Analysis of co-expression of these surface markers revealed a 1.4-fold increase in the number of CD44+CD25– (DN1) cells in Tg mice compared to WT (21.98% vs 15.7%) (Figure 2E, 2F). Taking into account the increase in CD3 expression on DN cells, this effect is compatible with the idea that expression of transgenic α-chain affects early differentiation of thymocytes, accelerating the appearance of TCR/CD3 complexes on the T cell membrane as soon as successful β-chain selection takes place [28, 29]. The number of DN2, DN3, and DN4 cells was similar in WT and Tg mice.

**Figure 3:**
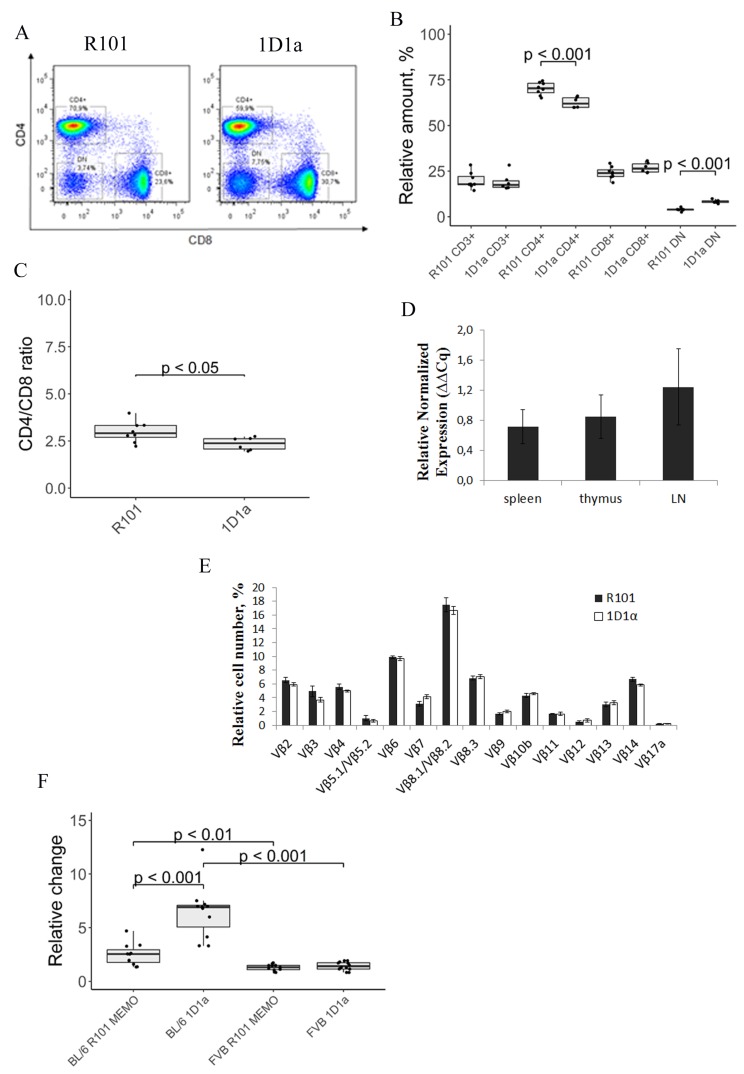
(**A**) Flow-cytometric analysis of CD8 and CD4 expression on CD3-gated lymphocytes from the spleen of wild-type (*left*) and Tg (*right*) mice. Data from one representative staining are shown. (**B**) The box plot represents the number of CD3, CD4, CD8, and DN cells in the spleen of R101 and 1D1α mice. (**C**) The ratio of CD4 to CD8 T cells in the spleen of R101 and transgenic (1D1α) mice is shown. (**D**) Relative expression of Tg T-cell receptor α-chain 1D1 in lymphoid organs of Tg mice. Four transgenes were used for the qPCR analysis. The spleen of one Tg mouse was used as a reference sample. *Tbp* and *ppia* were used as reference genes for normalization of gene expression. The samples were run in triplicate. The data represent the mean and sd. (**E**) Analysis of expression of 14 distinct Vβ gene families in peripheral blood T cells of Tg and WT mice. Six transgenes and 5 WT mice were used in the experiment. The data represent the mean ± sd. (**F**) Proliferative response of splenocytes to specific and third-party alloantigens. Cells from spleens of 3-month-old Tg and WT mice were used as responders. Splenocytes from B10.D2(R101) (syngeneic), C57BL/6 (specific alloantigen), and FVB (third-party alloantigen) were used as stimulators. All stimulators were treated with mitomycin C. The background proliferation (i.e. the proliferative response to syngeneic stimulators) was subtracted from values obtained in response to the specific and third-party alloantigens. The excess of proliferative response of splenocytes to C57BL/6 and FVB stimulators of R101 MEMO (BL/6 R101 MEMO and FVB R101 MEMO, respectively, and Tg (BL/6 1D1α and FVB 1D1α, respectively) over R101 splenocytes is shown.

To evaluate possible effects of transgenic α-chain expression on T cell commitment, we analyzed the pool of peripheral lymphocytes in Tg and WT mice. Figure 3A, 3B shows the expression of co-receptors CD4 and CD8 on the surface of CD3 cells in the spleen. Two-fold increase in the number of CD8–CD4– (DN) T cells and 1.12-fold decrease in the number of CD4 T cells were observed in the spleen of the Tg mice. Note that the number of CD3 and CD8 T cells was comparable in both types of mice. The ratio of CD4 and CD8 T cells was slightly but significantly (*p ˂* 0.05), higher in the spleen of WT mice (Figure 3C). These data show the minimum effect of transgenic α-chain expression on the ratio of CD4 and CD8 T cells.

Expression of transgenic α-chain TCRs could also affect homeostasis of the T cell repertoire in transgenic mice [23]. The activation phenotype of CD3+ splenocytes in Tg and WT mice will be discussed later.

As mentioned above, Tg T-cell receptor 1D1α corresponds to the Vα 11.3 allele, and there are no commercially available antibodies to detect this allele. So, we analyzed its expression in our Tg mice by qPCR. Because the nucleotide sequence of the forward primer overlaps with the CDR3 region of Tg TCRα, the resulting PCR product matches only the Tg α-chain 1D1.

We performed analysis of Tg α-chain expression in lymphoid organs of 1D1α mouse spleen, thymus, and lymphatic nodes (LNs). It was shown by qPCR that the amount of Tg α-chain mRNA was comparable in the tested organs (Figure 3D). Note that we were unable to detect 1D1α expression in the organs of the WT mice.

Because the specific α-chain could exhibit selectivity in pairing with particular members of Vβ-chain families, resulting in changes in TCR repertoire diversity, we examined the TCRβ repertoire in the peripheral blood of the transgenic mice. Analysis of 14 different Vβ families revealed no strong bias in Vβ usage in the Tg mice (Figure 3E). So, we assumed that TCRα 1D1α is able to pair with different endogenous β-chains.

To assess the response of the Tg cells to the specific antigen, we performed mixed lymphocyte reaction (MLR) using splenocytes from C57BL/6 mice (H-2b) and from FVB mice (H-2q) as specific and third-party stimulators, respectively. Syngeneic stimulator cells from B10.D2(R101) (K(d)I(d)D(b)) mice were used for measuring the background proliferation. All the stimulators were treated with mitomycin C. We used WT mice immunized with EL-4 cell line two months earlier (R101 MEMO) as a positive control as they had developed memory T cells specific to alloantigen H-2K^b^. We observed a significant increase in proliferation of the Tg cells in response to the specific alloantigen (H-2K^b^) compared to cells from both WT and R101 MEMO mice (Figure 3F). Note that all splenocytes stimulated with third-party alloantigen (H-2q) showed the same proliferation response.

We also performed MLR with stimulators treated with heat shock, because we had previously shown that stimulators exposed to severe heat shock (45°C, 1h) could induce selective response of memory T cells [30] (Supplementary text, Supplementary Figure 1B).

### Elimination of EL-4 tumor cells in 1D1α transgenic mice

**Figure 4:**
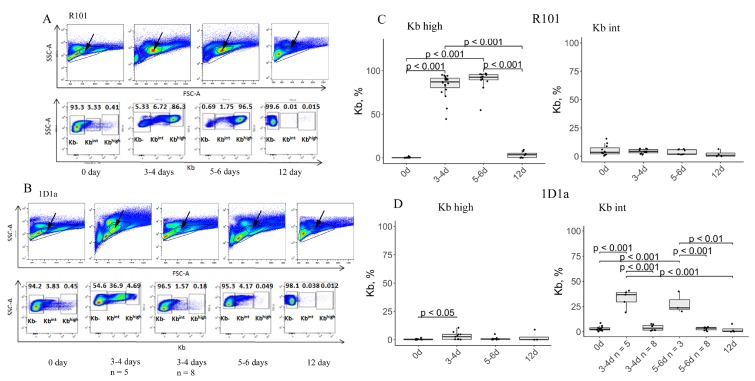
Estimation of tumor cell number in the peritoneal cavity on days 0, 3-4, 5-6, and 12 after immunization. (**A**, **B**) Flow-cytometric analysis of EL-4 tumor cells in the lavage of WT (**A**) and Tg (**B**) mice. Arrows on the upper panels indicate the presence of EL-4 cells in samples as determined by forward scatter (FSC) and side scatter (SSC). The lower panels represent the number of Kb positive cells in the peritoneal cavity. We distinguished two populations of Kb+ cells – with high (Kb^high^) or decreased (Kb^int^, intermediate) expression of Kb. Data from one representative staining are shown. (**C**, **D**) Box plots show the number of Kb^high^ (*left*) and Kb^int^ (*right*) EL-4 cells in the lavage of WT (**C**) and Tg (**D**) mice on the days 0, 3-4, 5-6, and 12 after immunization.

TCR containing the studied α-chain was initially isolated from memory T-cell hybridoma 1D1 that was obtained during the primary *in vivo* immune response of B10.D2(R101) mice to allogenic EL-4 tumor cells, followed by *in vitro* re-stimulation [22]. Consequently, this TCR is specific to MHC class I H-2K^b^. So, our next step was to analyze whether the expression of the Tg α-chain paired with random endogenous β-chains could influence the rejection of EL-4 cells. A number of EL-4 cells (10^7^) were injected into the peritoneal cavity of the Tg and WT mice. On days 0, 3-4, 5-6, and 12 after immunization, we performed flow-cytometric analysis of peritoneal lavage to establish the number of tumor cells (Kb positive). As expected, WT mice rejected the EL-4 tumor cells in 12 days (Figure 4A, 4C). In other words, 100% of studied WT mice cleared the tumor cells from the peritoneal cavity at day 12 after immunization (Supplementary Figure 2A). Approximately one half of the Tg mice (*n =* 8) were able to fully eliminate tumor cells from the peritoneal cavity on days 3-4 after immunization. Surprisingly, we discovered that the expression of the Kb molecule was decreased in EL-4 cells in the rest of the 1D1α mice (*n =* 5), whereas tumor cells were not eliminated from the peritoneal cavity (Figure 4B, 4D). Notice that the percent of Kb^int^ cells in these Tg mice (*n =* 5) was lower than the percent of Kb^high^ cells in WT mice (33.02 ± 8.8 vs 83.07 ± 13.37) on days 3-4 after immunization. On days 5-6, almost all of the analyzed Tg mice rejected the tumor cells (8 mice vs 3 mice with Kb^int^). To be sure that Kb negative EL-4 cells were indeed eliminated from the peritoneal cavity, we also monitored the presence/absence of tumor cells by parameters of forward scatter and side scatter (Figure 4A, 4B, *upper panels*). A survival curve demonstrated that the number of tumor bearing Tg mice was 38.5% and 27.3% at days 3-4 and 5-6 after immunization, respectively (Supplementary Figure 2A). Again, we should point out that these tumor cells had a decreased level of Kb expression. On the day 12 after immunization, 100% of the Tg mice had cleared all the tumor cells from the peritoneal cavity.

**Figure 5:**
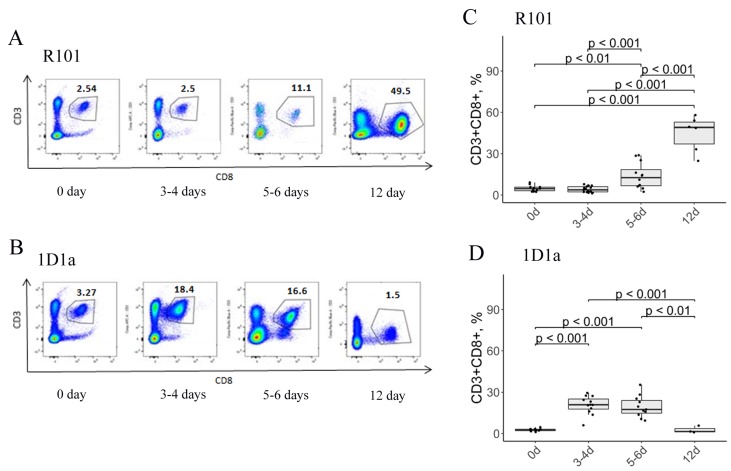
Flow-cytometric analysis of CD3/CD8 expression on lymphocytes from the peritoneal cavity of WT (A, C) and Tg (B, D) mice on days 0, 3-4, 5-6, and 12 after immunization. (**A**, **B**) Data from one representative staining are shown. (**C**, **D**) Box plots are used to visualize the distribution of a dataset.

Because CD8 T lymphocytes are the major type of cells responsible for killing tumor cells, we assessed the number of CD8 T cells in the peritoneal cavity on days 3-4, 5-6, and 12 after immunization and compared it with the level of cytotoxic T lymphocytes (CTLs) in peritoneal lavage of the intact mice (both Tg and WT mice that were not immunized with EL-4 cells, 0d after immunization). As expected, the elimination of EL-4 cells was accompanied by an elevated level of CD8 T cells in the peritoneal cavity of the WT mice – the number of CD8 T cells was up to 44.7% on day 12 after immunization (Figure 5A, 5C). Interestingly, we found different dynamics of CD8 cell recruitment in the peritoneal cavity of the Tg 1D1α mice during the immune response to EL-4. The peak level of CD8 T cells was about 20.7% and 19.5% on days 3-4 and 5-6 after immunization, respectively, in the peritoneal lavage of the Tg mice (1D1α) (Figure 5B, 5D). The number of CD8 T cells on day 12 in the Tg mice was comparable to the intact Tg control. These results are in strict compliance with the dynamics of EL-4 rejection in 1D1α Tg mice. Notice that the number of CD8 T cells in the lavage of the intact controls was comparable in WT and Tg mice (4.8% and 2.7%, respectively). So, at the peak of immune response to EL-4 WT mice recruited 2-fold greater number of CD8 T cells than the Tg mice.

Next we analyzed the dynamics of EL-4 rechallenge in R101 MEMO. As expected, we could barely detect EL-4 cells in lavage of the peritoneal cavity of R101 MEMO mice 3 days after immunization (Supplementary Figure 3A, *left*). The numbers of CD8 T cells in the R101 MEMO mice were about 31.6% and 34.6% on days 3–4 and 5–6, respectively (Supplementary Figure 3A, *right*). Note that the level of CD8 T cells in the peritoneal cavity of the intact R101 MEMO mice (0d after immunization) was 14.3%, which is significantly higher than in the intact WT and Tg mice.

**Figure 6:**
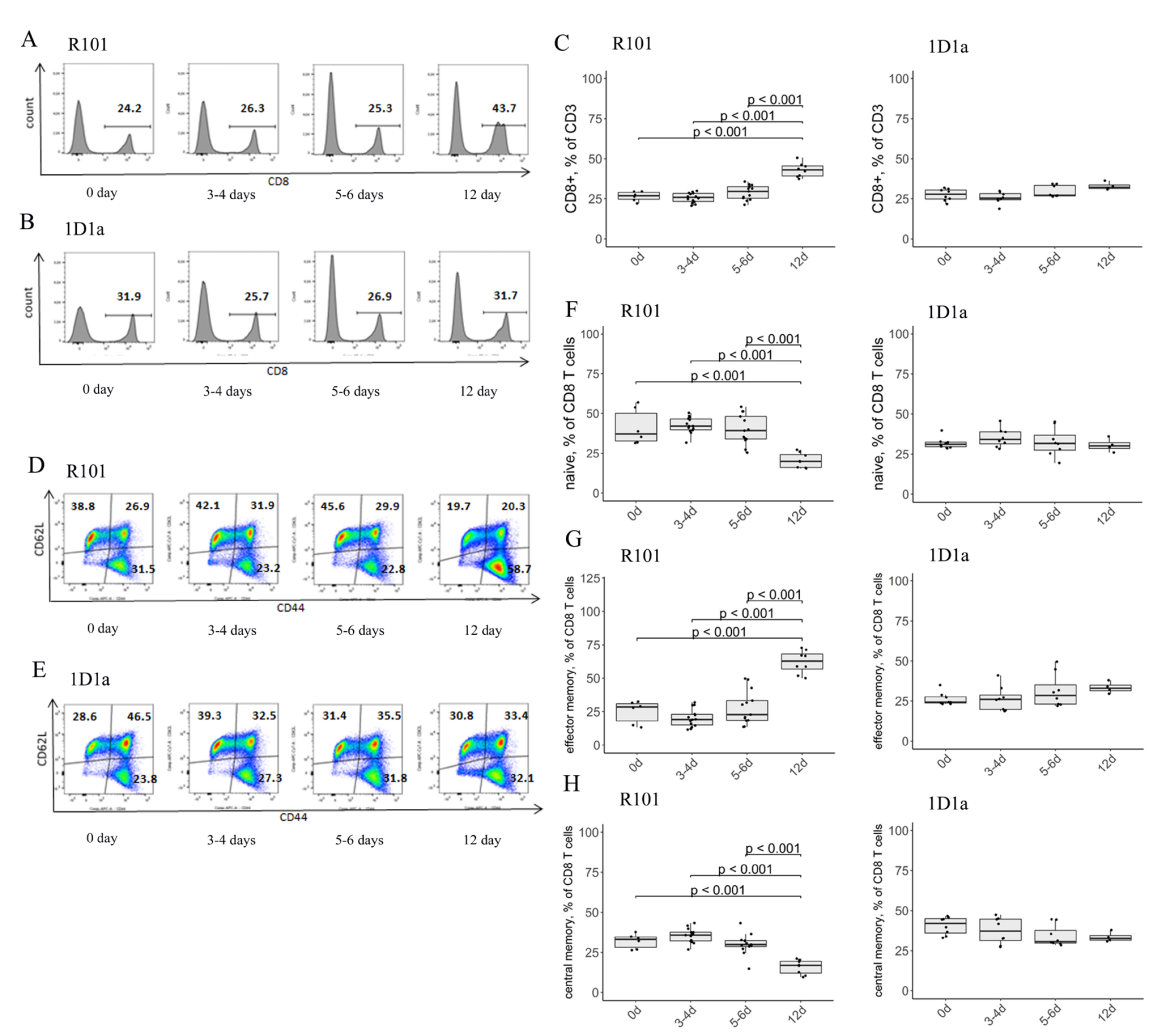
(**A**–**C**) Flow-cytometric analysis of expression of CD8 and activation markers CD44 and CD62L on splenic T lymphocytes on days 0, 3-4, 5-6, and 12 after immunization. The expression of CD8- on CD3-gated lymphocytes was defined in the spleen of WT (A, C) and Tg (**B**, **C**) mice. (A, B) Data from one representative staining are shown. (**C**) The box plot shows the relative number of CD8+ T cells in the spleen of R101 (*left*) and 1D1α (*right*) mice. (**D**–**H**) Flow-cytometric analysis of co-expression of CD44 and CD62L markers on the surface of the CD8 subset of T lymphocytes in the spleen of WT (D, F, G, H) and Tg (E–H) mice. Data from one representative staining are shown. The box plots show the distribution of cells with naive (CD44-CD62L+) (F), effector memory (CD44+CD62L–) (**G**), and central memory (CD44+CD62L+) (H) phenotypes.

We also analyzed T-cell subpopulations in the spleen of the WT and Tg mice on days 3-4, 5-6, and 12 after immunization. We observed a slight but significant increase in CD3 expression in the spleen of the WT and Tg mice on day 12 after immunization as compared to the intact WT and Tg control, respectively (Supplementary Figure 4). As expected, a 1.6-fold increase in the number of CD8 T cells was determined on day 12 after immunization in the WT mice compared to intact WT control (0d after immunization) (Figure 6A, 6C). Flow-cytometric analysis of co-expression of CD44 and CD62L markers, which define the activation phenotype, revealed a 2.5-fold increase in CD8 effector memory T cells along with naive and central memory CD8 T cells decreasing 2-fold on day 12 after immunization in the WT mice compared to the intact WT control (Figure 6D, 6F–6H). These results are in accordance with tumor rejection data. We were unable to detect any significant changes in the number of CD8 T cells as well as effector memory, central memory, and naive CD8 T cells in the spleen of the Tg mice on any of the indicated days compared to the intact Tg controls (0d after immunization) (Figure 6B, 6E–6H). We should note that initially the number of central memory CD8 T cells was higher in the Tg mice compared both to the WT and R101 MEMO mice (40.55% vs 32%, and 28.5%, respectively).

Flow-cytometric analysis of T cell subpopulations in the spleen of R101 MEMO mice on days 3-4 and 5-6 after immunization revealed a slight 1.25-fold increase and 1.2-fold increase in the number of CD3 and CD8 subpopulation, respectively, on days 3-4 and 5-6, respectively, compared to intact R101 MEMO control (Supplementary Figure 3B). We found a 1.7-fold increase in the number of CD8 effector memory T cells along with a 1.3-fold decrease in naive CD8 T cells on days 5-6 after immunization (Supplementary Figure 3C). We observed no changes in the number of central memory CD8 T cells in the spleen of immunized R101 MEMO mice on any of the indicated days (Supplementary Figure 3C).

### Adoptive transfer (AT) of transgenic cells and cells transduced with 1D1α

Further, we were interested whether 1D1α-expressing cells have enough capacity to accelerate elimination of EL-4 tumor cells from WT mice with adoptively transferred transgenic or transduced cells (Supplementary text, Supplementary Figure 5, 6). We found that indeed Tg splenocytes and T cells transduced with 1D1α adoptively transferred (AT) into WT mice maintained their functionality during 14 days after AT, and such mice were able to eliminate tumor cells from their peritoneal cavity in 6 days after immunization of the mice with EL-4 cells compared to control mice that did not receive any cells harboring 1D1α. A survival curve showed that when immunization was performed no longer than at 14d after the intraperitoneal AT of Tg and 1D1α-transduced cells (0d, 7d, 14d) 77-100% and 37.5-100% of the experimental mice cleared the tumor cells, respectively (Supplementary Figure 2B, 2C). When intravenous AT was performed and mice were immunized 0d and 7d after the AT, 100% of the experimental mice were able to eliminate the EL-4 cells (Supplementary Figure 2D).

Modified T cells are in most demand for adoptive transfer therapy, particularly in cancer treatment. Maintaining the ability of both Tg and transduced cells adoptively transferred into the WT mice to kill the tumor is significant for potential application in the tumor therapy.

## DISCUSSION

In this study, we presented TCRα Tg mice 1D1α. The α-chain was isolated from the memory T-cell hybridoma 1D1 specific to the MHC class I H-2K^b^. We did not observe impairment of intrathymic development of T cells in the Tg mice. Analysis of subpopulations revealed an increase in the DN cells in the thymus and DN T cells in the spleen of the 1D1α mice. These results are in accordance with others studies – due to premature TCRα expression an elevated number of DN cells develop in most TCRα transgenic mice [29, 31, 32]. We also demonstrated no preference in the TCR Vβ usage in CD3 lymphocytes in naive Tg mice. In MLR experiments, we demonstrated that lymphocytes harboring Tg TCRα paired with random endogenous β-chains showed enhanced proliferation response to specific stimulators.

Using these single TCRα Tg mice, we estimated the elimination dynamics of tumor cells bearing H-2K^b^ (EL-4). The 1D1α Tg mice were able to reject EL-4 cells within 3-6 days, whereas in WT mice it required 12 days. This means that Tg mice possess more T cells with TCRs reactive to the antigen and indicates that TCRα plays the dominant role in specific antigen recognition. A similar result was obtained by another group of scientists [19]. Using single TCRα Tg mice, they also demonstrated that antigen recognition appeared to be mediated mainly by the α-chain. We should note that the dynamics of lymphoma cell elimination by the Tg mice was similar to that in the R101 MEMO mice, which had already developed CD8 memory T cells to the antigen. Earlier it was observed that memory T cells stimulated with specific antigen could proliferate faster than naive T cells *in vivo* [33, 34]. We also performed *in vitro* experiments to confirm the *in vivo* result. Activated splenocytes that indeed had their own TCRαβ receptors were transduced with α-chain 1D1. Such lymphocytes were able to kill lymphoma cells *in vitro*, whereas non-transduced cells had no influence on EL-4 cells growth. Moreover, it was demonstrated that transgenic and 1D1α transduced cells adoptively transferred into WT mice maintained their capacity to rapidly eliminate EL-4 cells from the peritoneal cavity of these mice. Both transgenic and transduced 1D1α T cells exhibited a profound therapeutic effect when adoptively transferred into naive WT mice simultaneously with EL-4 cell transplantation. Moreover, adoptively transferred transgenic and transduced 1D1α T cells persisted in a naive host for 14 days post-transfer and retained their ability to rapidly eliminate the specific tumor cells. These findings are of particular importance as long-term persistence and functional activity of transferred T lymphocytes affect the overall efficiency of adoptive transfer therapy in cancer [35, 36]. These data demonstrate that T lymphocytes of a naive host transduced with a single TCRα of tumor-specific memory T cells can significantly improve the anti-tumor immunity of the host.

Analysis of Kb positive cells (EL-4) in the peritoneal lavage of immunized Tg mice revealed that on days 3-4 after immunization half of the mice had killed all the tumor cells, whereas EL-4 cells in the rest of the Tg mice were not fully eliminated – 33% of the analyzed cells were Kb positive. The main surprise was that these cells expressed less Kb than EL-4 cells from WT lavage or cultured alone. We suggest two possible explanations of these data. 1. Tumor cells are heterogeneous and initially have at least two populations expressing high and intermediate levels of Kb. Our data suggest evidence for much faster elimination of Kb^high^ tumor cells in all the transgenic mice than of Kb^int^ cells. In other words, at the time of analysis (3-6 days after immunization) we see the result of immune selection in the transgenic mice, during which their immune system eliminates Kb^high^ tumor cells. However, we can still observe Kb^int^ cells at days 3-6 after immunization in some Tg mice (Figure 4B, 4D). On the contrary, the immune response in the WT mice began later than 3-6 days after immunization, and at day 12 of analysis all EL-4 cells were eliminated from the peritoneal cavity. 2. We can assume that tumor cells have enhanced immune escape potential in 1D1α mice. The decrease in Kb expression could probably lead to the loss of MHC by tumor cells, which is known as a common event in the escape stage of tumor progression [37–39]. Earlier, we showed that 30 days after immunization EL-4 cells lost all their Kb molecules in TCRβ Tg mice [24]. But as the repertoire of TCRs specific to the alloantigen is much larger in TCRα Tg mice than in TCRβ Tg mice, these mice were able to fully eliminate lymphoma cells before EL-4 cells could lose all their MHC molecules.

Once immunized with lymphoma cells, the WT mice developed more cells with effector phenotype in their spleen and recruited CD8 T cells into the tumor injection site. This is a common sequence of the primary immune response [40, 41]. Memory CD8 T cells can be found directly in the tissues where they provide defense against secondary infection [42, 43]. According to these data, the intact R101 MEMO mice initially had more CD8 T cells in the peritoneal lavage than the intact WT mice (14.3% vs 4.8%). So, as expected, the R101 MEMO mice re-immunized with EL-4 could rapidly recruit significantly fewer CD8 T cells into the peritoneal cavity than immunized WT mice – 20% at the peak of immune response on days 3-6 vs 40% on day 12, respectively. Intact 1D1α Tg mice initially had approximately the same number of CD8 T cells in the peritoneal cavity as WT mice (2.7%), but in 3-6 days after immunization the recruited number of CD8 T lymphocytes was similar to that in immunized R101 MEMO mice (about 18%). Notice that the total numbers of CD8 T cells after immunization in R101 MEMO and WT mice were 31-34% and 45% on days 3-6 and 12, respectively, but in TCRα Tg mice it was approximately 20% on days 3-6 after immunization. This suggests that TCRα Tg cells possess higher potential for rapid killing of EL-4 cells than even the specific memory T cells.

The fact that initially the Tg mice had an increased ratio CD8/CD4 and more CD8 T cells with the phenotype of central memory cells may be the reason for the rapid immune response to the EL-4 cells and lack of prominent differences in the dynamics of both naive and effector memory CD8 T cells during the response. As proposed earlier, central memory T cells could mediate the development of reactive memory cells and are ready to proliferate and differentiate into effectors upon antigenic stimulation [44]. This is confirmed by our finding that in the spleen of the Tg 1D1α mice the accumulation of effector CD8 cells on day 12 post-immunization was accompanied with a decrease in the relative number of central memory T cells (Figure 6).

Together, our results show that single TCRα Tg mice can eliminate lymphoma cells harboring specific alloantigen as quickly as R101 MEMO mice that had already developed specific memory T cells. Moreover, the Tg mice require fewer CD8 T cells to deal successfully with this task than either WT or R101 MEMO mice. These findings suggest an instructive role of TCR in development and functioning of T lymphocytes and may provide the possibility for fast identification of dominant α-chains of TCR for subsequent enhancement of immunity. Our alternative therapeutic approach based on chain centricity of the TCRs has several important benefits compared to existing strategies, and it potentially could be used in tumor adoptive cell therapy.

## MATERIALS AND METHODS

This study was carried out in strict accordance with the recommendations in the *Guide for the Care and Use of Laboratory Animals* of the National Institutes of Health (USA). The protocol was approved by the *Committee on the Ethics of Animal Experiments* of the N. N. Blokhin Cancer Research Center, Moscow, Russia.

### Animals

Mice of C57BL/6 (H-2b), C57BL/10 (H-2b), B10.D2(R101) (H-2g1, Kd I-Ad I-Ed Db), and FVB (H-2q) strains were obtained from the breeding facility of the N. N. Blokhin Cancer Research Center. The F1 hybrid (CBA/Lac × C57BL/6) mice were purchased from the “Stolbovaya” nursery (Pushchino, Moscow Region, Russia). The Tg mice expressing the TCR α-chain of the memory hybridoma 1D1 (1D1α) were generated in the Laboratory of Transgenesis, Institute of Gene Biology, Russian Academy of Sciences, and bred in the Laboratory of Regulatory Mechanisms in Immunity, N. N. Blokhin Cancer Research Center. All mice used in the experiments were 3-6 months old. In each set of experiments mice of the same gender were used.

Genotyping of the 1D1α Tg mice was performed using the following primers: forward 5′-ccagctcgaggac aggggccatg-3′; reverse 5′-aacaccgcggtctgtctcagagtgt-3′. Primers specific to the mouse CD8 gene were used as a control during PCR: CD8 forward 5′- cgaactccgaatctttcc aaa-3′ CD8 reverse 5′-tacttattattcgtgtccctca-3′. Each generation of Tg mice were carefully analyzed for the presence of the Tg T cell receptor α-chain 1D1. All the experiments were carried out using selected Tg mice.

### Cloning cDNA encoding the α-chain of memory TCR

Full-length α-chain from 1D1 T-cell hybridoma was cloned into the pTα cassette using the XmaI and NotI restriction sites as described in the original paper [26].

### Cell lines

EL-4 lymphoma cells were transplanted intraperitoneally (i.p.) in syngeneic C57BL/6 mice at (3-5) × 10^6^ per mouse and grown as ascites tumors. The HEK 293T cell line was cultured in DMEM medium supplemented with 4.5 g/L glucose (Sigma, USA) and 10% FCS (GIBCO BRL, USA).

### Immunization

B10.D2(R101) (WT) mice were immunized i.p. with 10^7^ EL-4 tumor cells per mouse to obtain memory T cells in 2 months after immunization (R101 MEMO mice). For EL-4 rejection experiments, WT, 1D1α, and R101 MEMO mice were i.p. injected with 10^7^ EL-4 cells. For adoptive transfer experiments, WT mice were i.p. injected with 5 x 10^5^ EL-4 tumor cells. On the indicated days, peritoneal lavages were collected and subjected to flow-cytometric analysis.

### Cells isolation

Lymphocytes were gently squeezed from the stroma of the mouse spleen and thymus in a Potter homogenizer with a conic pestle. Blood samples were collected from the retro-orbital venous sinus of the WT and 1D1α Tg mice. Erythrocytes were lysed with RBC lysis buffer (BioLegend, USA) and the cells were washed with PBS (200g, 5 min, 4°C).

### Mixed lymphocyte reaction

Lymphocytes were gently squeezed from the stroma of mouse spleen in a Potter homogenizer with a conic pestle. Erythrocytes were lysed with lysis buffer. Stimulators were treated with mitomycin C (25 mg/ml, 30 min, 37°C) and then washed 3 times with PBS. The responders and stimulators were seeded in 96-well plates at ratio 3:5 in RPMI-1640 medium (GIBCO BRL, Grand Island, NY, USA) supplemented with 10% FCS, 50 mM 2-ME (Merck, Darmstadt, Germany), and antibiotic Ciprofloxacinum (KRKA, Novo Mesto, Slovenia). Acute heat shock was induced by incubation of the splenocytes at 45°C for 60 min. Proliferation was measured by [^3^H]-thymidine (Saint-Petersburg “Izotop”, Saint-Petersburg, Russia) incorporation after a 3-day co-incubation.

### Antibodies

Samples were stained with the following antibodies: FITC-, eFluor450-, and APC-conjugated anti-CD8a (Clone 53-6.7, eBioscience, San Diego, CA, USA), FITC conjugated anti-CD4 (Clone GK1.5, eBioscience), APC/Cy7-conjugated anti-CD62L (Clone MEL-14, BioLegend, San Diego, CA, USA), APC-conjugated anti-CD44 (Clone IM7, eBioscience), Alexa Fluor 647- and eFluor 450-conjugated anti-CD3 (Clone 17A2, eBioscience), FITC- and PE-conjugated anti-Kb (Clone AF6-88.5.5.3, eBioscience), FITC-conjugated anti-CD25 (Clone 3C7, BD Pharmingen, USA), and FITC-conjugated Mouse Vβ TCR Screening Panel (BD Pharmingen).

### Flow cytometry

The cells were stained with antibodies at 4°C for 40 min. Analysis was performed on a BD FACSCanto II flow cytometer using the BD FACSDiva 6.0 program. Dead cells were excluded from the analysis via staining with propidium iodide (PI, Sigma, USA) and measuring the forward and side scattering. The results were analyzed using Flow Jo 7.6.

### Total RNA isolation from organs

Each organ was powdered using liquid nitrogen. Total RNA isolation was performed using TRI reagent (MRC, Inc, TR118).

### Reverse transcription and real-time PCR

RNA was treated with DNaseI (Thermo Fisher Scientific EN0521). cDNA synthesis was performed using RevertAid First Strand cDNA Synthesis Kit (Thermo Scientific, #K1622). The following primers specific to murine genes were used: 1D1α forward 5′-ttctgtgctgctgatctcatgt-3′, reverse 5′-caggcagagggtgc tgtc-3′, hprt forward 5′-aactttgctttccctggtt-3′, reverse 5′-cgctcatcttaggctttgt**-**3′, ppia forward 5′-gactgaatgg ctggatgg-3′, reverse 3′-cagaaggaatggtttgatgg-3′. The qPCR results were analyzed using CFX Manager Software for qPCR data (BioRad, USA).

### Plasmids and transfection

We cloned TCRα 1D1 and TCRα 1D1 fused to GFP into MigRI retroviral vector using the AgeI and SalI restriction sites. As a packaging plasmid for the retroviruses, we used pCL-Eco (a kind gift of Beliavskiĭ AV). Calcium-phosphate transfection was performed to deliver the plasmids into the 293T packaging cell line.

### Transduction

We collected viruses 48h (first portion) and 72h (second portion) after transfection. The day before transduction, splenocytes were isolated as described above and activated with ConA (3 μg/ml, Sigma, USA) and murine IL-2 (10 U/ml, Sigma) for 24h. Retroviral transduction was performed by two rounds of spinoculation using the first portion of the virus in the first round and using the second portion of the virus in the second round. The conditions for each spinoculation were the following: 2h, 2000g, 22°C. The efficiency of transduction was estimated 3 days after by measuring GFP fluorescence using flow cytometry, and on average it was 40-60%. The cells were immediately used in the experiments 3-4 days after transduction. Cells with transduction efficiency lower than 30% were not used in the experiments.

### *In vitro* experiments

Splenocytes were activated and transduced with 1D1α chain. Three to four days after transduction, the lymphocytes were mixed in culture with EL-4 cells at ratio 2:1. 24h, and after co-culturing the cells were analyzed by flow cytometry using the antibodies specific to the Kb molecule and anti-CD3, CD4, and -CD8 antibodies.

### Adoptive transfer

5 × 10^6^ or 10 × 10^6^ Tg splenocytes and 5**´**10^6^ cells transduced with 1D1α chain were adoptively transferred into the peritoneal cavity of R101 mice. 20**´**10^6^ Tg LNs cells and 5 × 10^6^ cells transduced with TCRα 1D1α were intravenously transferred into R101 mice. Immunization with EL-4 cells was performed 0, 7, 14, and 28 days (unless otherwise specified) after the transfer. Six days after immunization, the percent of Kb positive cells was estimated in the peritoneal cavity of the mice using flow cytometry.

### Statistical analysis

All data are presented as mean ± sd. To determine statistical significance, *p* values were calculated using ANOVA RStudio.

## SUPPLEMENTARY MATERIALS



## Abbreviations

CDR: Complementarity-determining region
CTLs: Cytotoxic T lymphocytes
DN: Double negative
DP: Double positive
GFP: Green fluorescent protein
HIV: Human immunodeficiency virus
LNs: Lymphatic nodes
MHC: Major histocompatibility complex
MLR: Mixed lymphocyte reaction
pMHC: peptide–MHC complex
qPCR: Quantitative polymerase chain reaction
SP: Single positive
TCR: T-cell receptor
Tg: Transgenic
WT: Wild-type
