# Supplementary material for: Dominant role of the α-chain in rejection of tumor cells bearing a specific alloantigen in TCRα transgenic mice and in in vitro experiments
Source: Oncotarget. 2019 Aug 6;10(47):4808–21. doi: 10.18632/oncotarget.27093 (PMC6690675; doi:10.18632/oncotarget.27093)
Supplement: Supplementary file 1 [file oncotarget-10-4808-s001.pdf]

Dominant role of the α-chain in rejection of tumor cells bearing a specific alloantigen in TCRα transgenic mice and in *in vitro* experiments

SUPPLEMENTARY MATERIALS

Characterization of transgenic 1D1α mice

We demonstrated that cells from R101 MEMO and 1D1α mice had two times higher indices of proliferation in response to heat shock-specific stimulators than those from WT mice (Supplementary Figure 1B). We were unable to detect any proliferation response to heat shocked third-party stimulators. Therefore, we can assume that Tg cells proliferating in response to heat shock stimulators are memory T cells.

Adoptive transfer of transgenic cells and cells transduced with 1D1α

The experiment was organized as follows: the specified amount of transgenic/transduced cells (referred to as "exp" in the figures) and WT cells (control) were adoptively transferred (AT) into R101 mice; 0, 7, 14, and 28 days after the transfer, immunization with thymoma cells was performed; on day 6 after immunization, we analyzed the number of Kb positive cells in the peritoneal lavage.

We found that Tg splenocytes intraperitoneally transferred into R101 mice (exp) have the capacity to fully eliminate EL-4 cells from the peritoneal cavity when immunization was performed 7 or 14 days after the transfer (Supplementary Figure 5A). Mice immunized 28 days after the transfer were unable to kill lymphoma cells on day 6 after immunization. When adoptive transfer and immunization were performed simultaneously (0d), two-thirds of the mice were able to completely eliminate EL-4 cells by day 6 after immunization. In other words, when AT and immunization were performed simultaneously, the number of tumor-bearing mice was 33.3%; when immunization was performed 7 or 14 days after the AT, 100% of the experimental mice could eliminate lymphoma cells from the peritoneal cavity (Supplementary Figure 2B). No experimental mice cleared the tumor cells when immunization was performed 28d after the AT. This result is not surprising as we have shown previously that some Tg mice eliminated EL-4 cells more slowly than other Tg mice but indeed faster than WT mice (Figure 4).

All mice with intraperitoneally adoptively transferred transduced splenocytes (exp) were able to eliminate lymphoma cells when immunization was performed 7 days after the transfer (Supplementary Figure 5B). When adoptive transfer and immunization were performed simultaneously, half of the mice (exp $n = 7$) rejected tumor cells by day 6 after immunization, while the other half (exp $n = 8$) could not. Notice that we detected a decrease of Kb expression in exp mice that did not reject EL-4 cells compared to control mice. When immunization was performed 14 days after the transfer, only three mice out of eight were unable to eliminate EL-4 cells from the peritoneal cavity. In addition, similarly to the result obtained with Tg transferred cells, mice immunized 28 days after the transfer of transduced cells could not reject lymphoma cells (Supplementary Figure 5B). The survival curve demonstrated that 46.7% of experimental mice cleared tumor cells when immunization and AT were performed simultaneously; 100% of experimental mice eliminated tumor cells when immunization was performed 7d after AT and, 37.5% of experimental mice cleared tumor cells when immunization was performed 14d after AT (Supplementary Figure 2C). Note that no experimental mice cleared the tumor cells when immunization was performed 28d after AT.

We also performed intravenous injection of transgenic LN cells (exp) into R101 mice and showed that such mice were able to eliminate EL-4 cells from their peritoneal cavity when immunization was performed 0d or 7d after the transfer. When immunization was performed 14d after AT, the experimental mice could not reject EL-4 cells, but the expression of Kb decreased (Supplementary Figure 6A). Again, mice immunized 28 days after the transfer of Tg LN cells could not reject the lymphoma cells. In other words, 100% of experimental mice could clear tumor cells from the peritoneal cavity when immunization was performed 0d and 7d after the AT and none of the experimental mice eliminated EL-4 cells when immunization was performed 14d (but tumor cells had decreased level of Kb expression) or 28d after AT (Supplementary Figure 2D).

The intravenous transfer of 1D1α transduced cells (exp) into R101 mice along with simultaneous immunization with EL-4 cells showed no differences in the capacity of such mice to reject the lymphoma cells (Supplementary Figure 6B). When the immunization was performed 7 days after the transfer, none of the experimental mice could also clear tumor cells, but the EL-4 cells showed a decreased level of Kb expression (Supplementary Figure 6B).

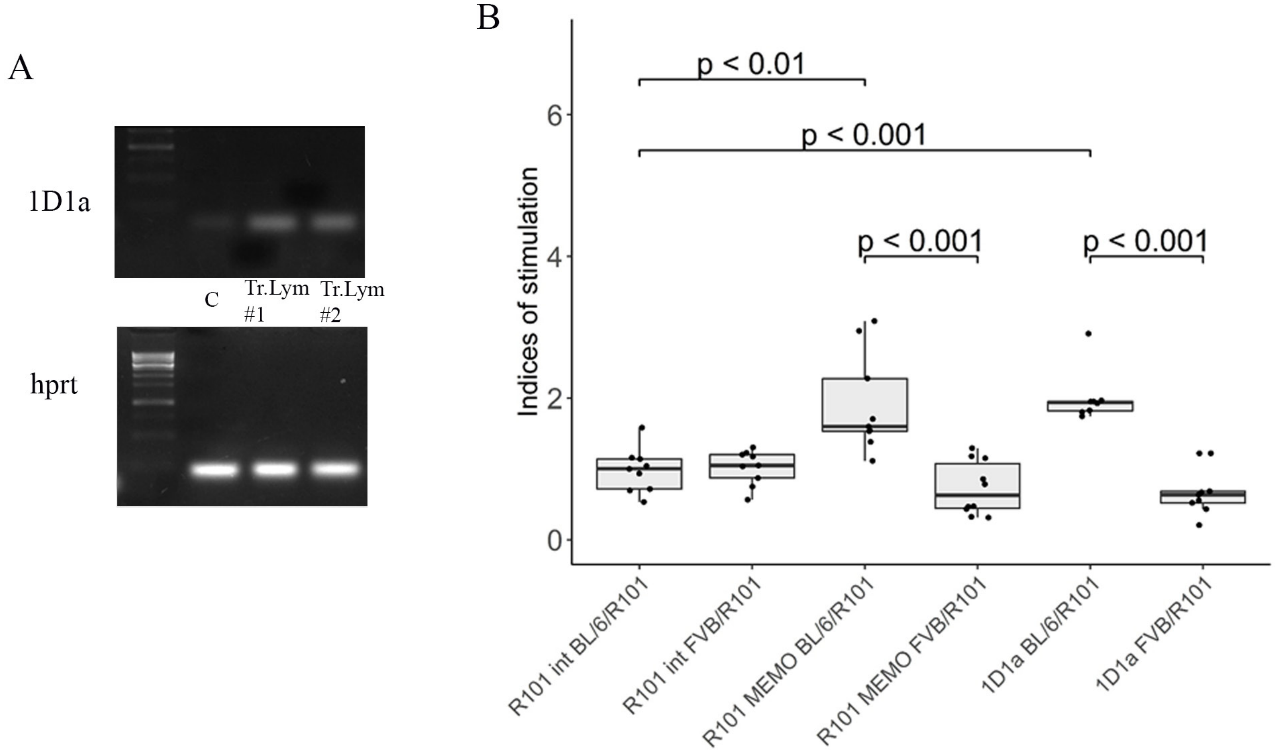

Supplementary Figure 1: (**A**) RT-PCR was used to confirm the presence of the Tg α-chain in transduced T cells 5 days after transduction. *Hprt* was used as a housekeeping gene control. Two samples of independently transduced T cells were analyzed. C – control un-transduced lymphocytes, Tr.Lym – transduced lymphocytes. (**B**) Splenocyte proliferative response to specific and third-party stimulators treated with severe heat shock (45°C, 1h). Cells from spleens of 3-month-old Tg and WT mice were used as responders. Splenocytes from B10.D2(R101) (syngeneic), C57BL/6 (specific alloantigen), and FVB (third-party alloantigen) were used as stimulators. The indices of stimulation (i.e. the excess of proliferative response of respective mice cultured with syngeneic stimulators over proliferative response developed during culturing with allogenic stimulators C57BL/6 and FVB) of splenocytes from intact R101 (R101 int BL/6/R101 and R101 int FVB/R101), R101 MEMO (R101 MEMO BL/6/R101, and R101 MEMO FVB/R101) and Tg (1D1α BL/6/R101 and 1D1α FVB/R101) mice are shown.

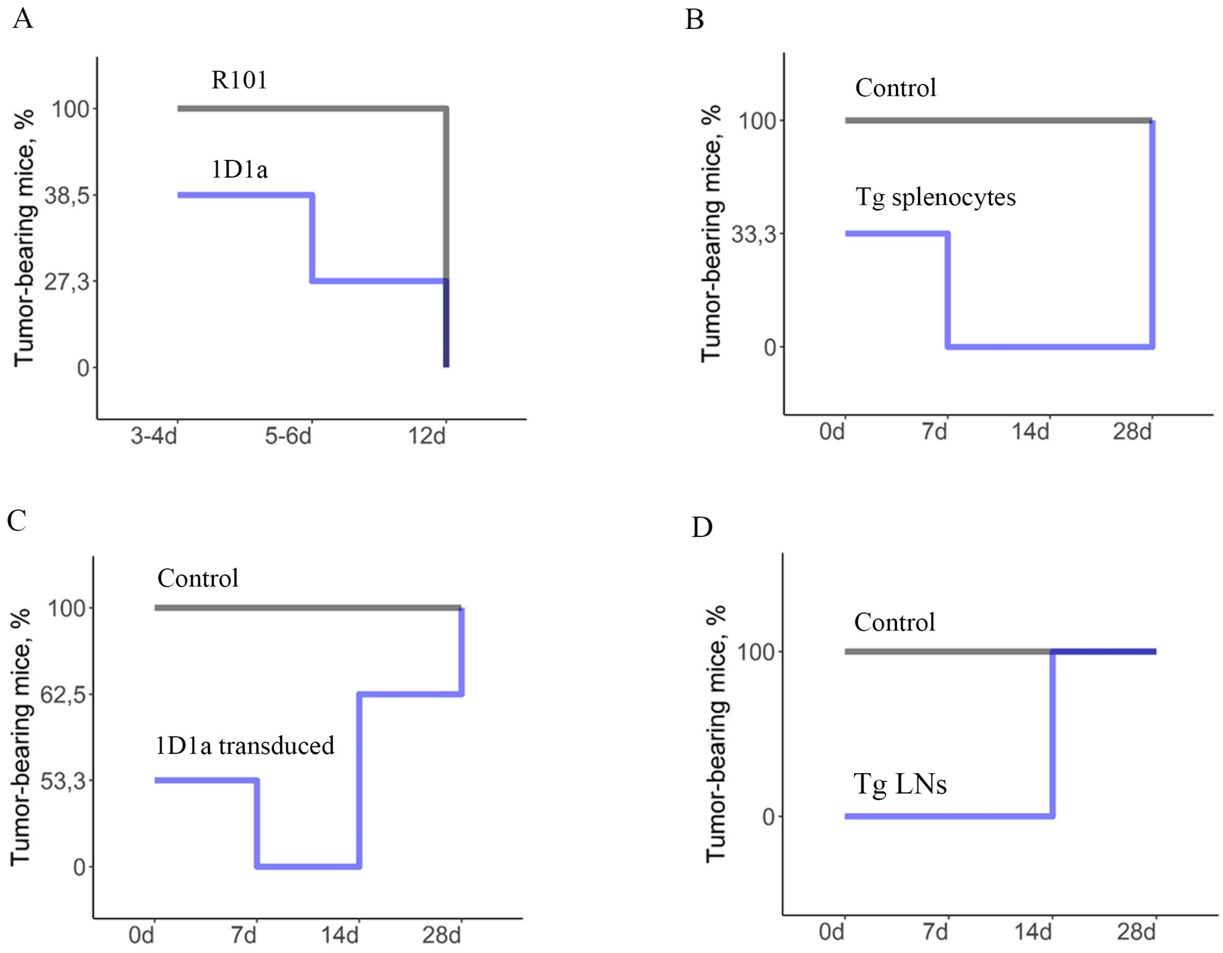

Supplementary Figure 2: (**A**) Survival curve shows the percent of WT (black line) and Tg (blue line) tumor-bearing mice on days 0, 3-4, 5-6, and 12 after immunization. (**B**–**D**) The percent of control (black line) and experimental (blue line) (mice with intraperitoneal adoptively transferred Tg splenocytes (B) and 1D1α-transduced T cells (C), and intravenous AT Tg LNs cells (D)) tumor-bearing mice is shown. Immunization was performed 0 d, 7 d, 14 d and 28 d after AT. The number of tumor cells in the peritoneal cavity was analyzed on day 6 after the immunization. Control – WT splenocytes/non-transduced T cells/WT LNs.

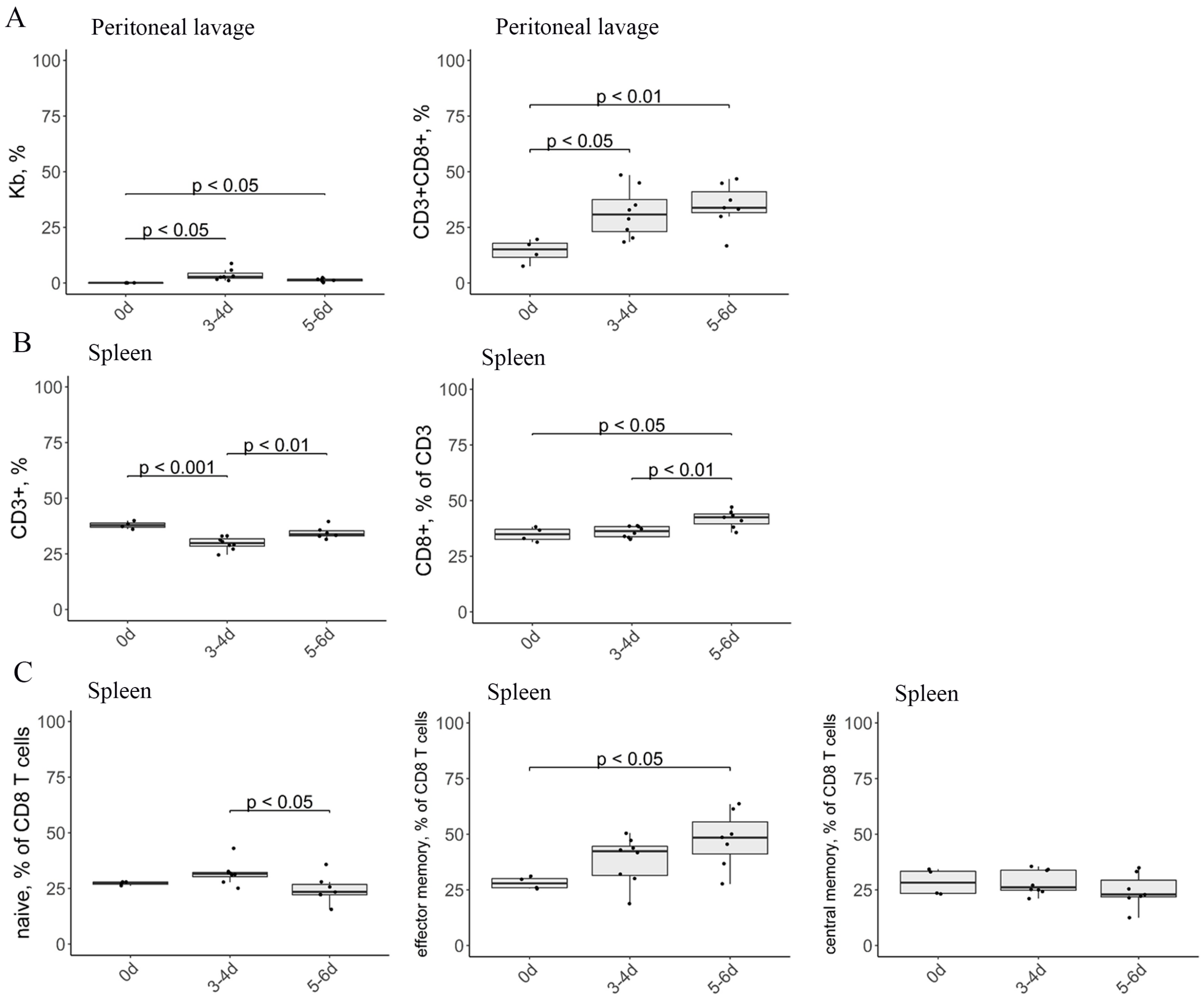

Supplementary Figure 3: (**A**) Number of Kb positive EL-4 tumor cells (*left*) and CD8 T cells (*right*) in the peritoneal cavity of R101 MEMO mice on days 0, 3-4, and 5-6 after immunization. (**B**) Box plots show the number of CD3+ cells (*left*) and expression of CD8 on CD3-gated lymphocytes (*right*) in the spleen of R101 MEMO mice. (**C**) Co-expression of CD44 and CD62L markers on the surface of the CD8 subset of T lymphocytes from the spleen of R101 MEMO mice was analyzed. Box plots show the distribution of cells with naive (CD44–CD62L+) (*left*), effector memory (CD44+CD62L–) (*middle*), and central memory (CD44+CD62L+) (*right*) phenotype.

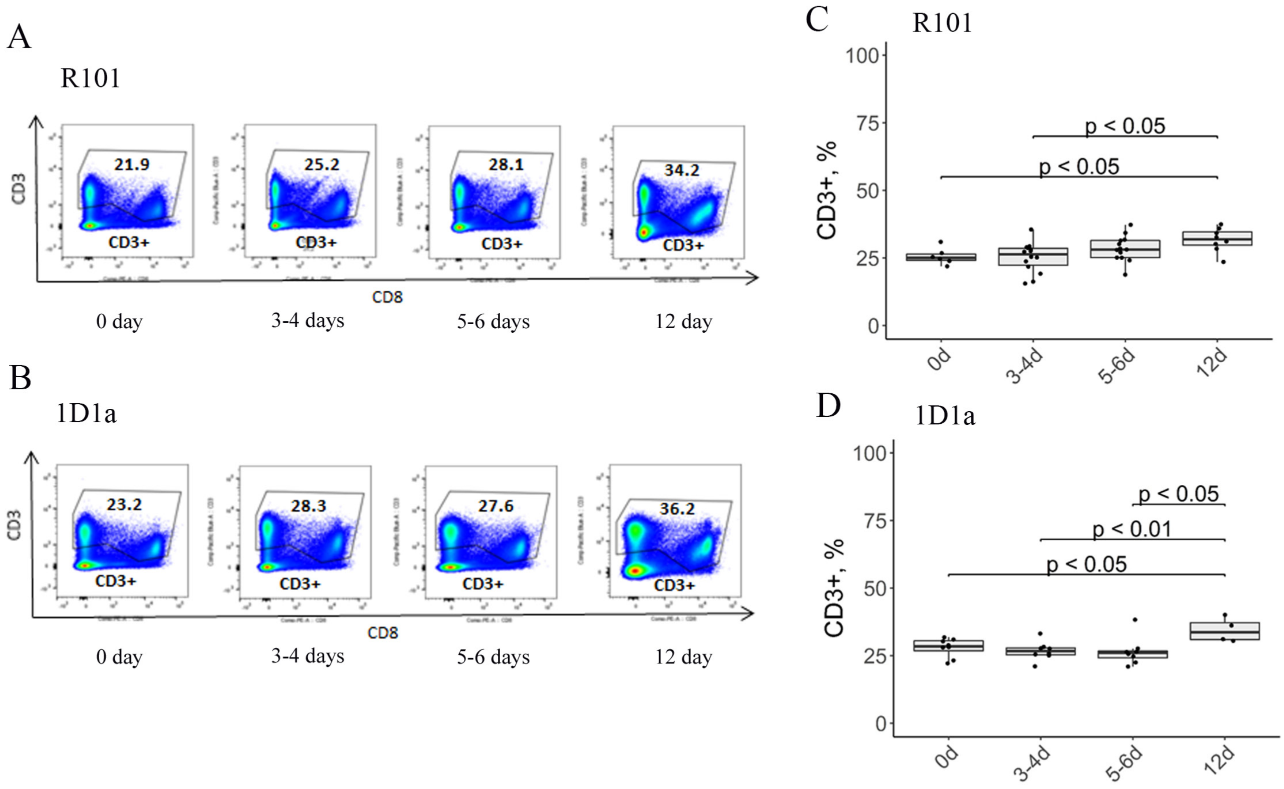

Supplementary Figure 4: Flow-cytometric analysis of CD3 expression on splenic lymphocytes of R101 (**A**) and Tg (**B**) mice on days 0, 3-4, 5-6, and 12 after immunization. Data from one representative staining are shown. The box plot shows the relative number of CD3+ T cells from the spleen of R101 (**C**) and 1D1α (**D**) mice.

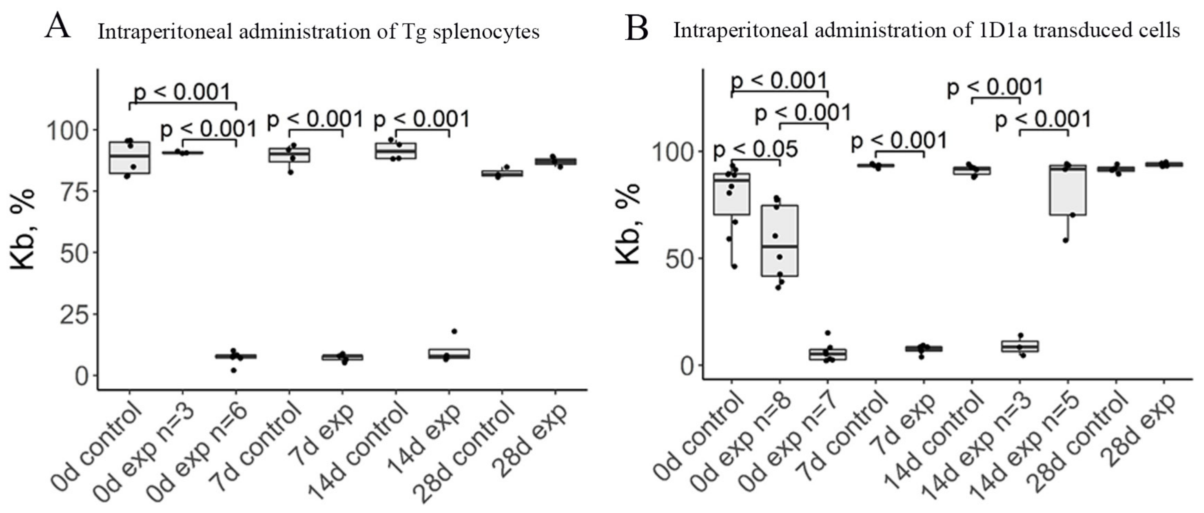

Supplementary Figure 5: Intraperitoneal adoptive transfer of transgenic cells (**A**) and cells transduced with 1D1α (**B**). (**A**) 10 × 10^6 splenocytes from WT and Tg mice were adoptively transferred into the peritoneal cavity of R101 mice simultaneously with immunization with EL-4 cells (0d control and 0d exp, respectively). 5 × 10^6 WT and Tg splenocytes were adoptively transferred into the peritoneal cavity of R101 mice along with immunization performed 7, 14, and 28 days after the transfer (7d, 14d, 28d control and 7d, 14d, 28d exp, respectively). (**B**) Splenocytes of WT mice were transduced with 1D1α chain and independently with GFP to estimate the transduction efficiency. The immunization with EL-4 lymphoma cells was performed 0d, 7d, 14d, and 28d after the transfer of 5 × 10^6 non-transduced or transduced cells into the peritoneal cavity of R101 mice (0d, 7d, 14d, 28d control and 0d, 7d, 14d, 28d exp, respectively). Analysis of the number of Kb positive cells in the peritoneal lavage was performed 6 days after immunization. Control - WT splenocytes/non-transduced T cells, exp (experiment) - Tg splenocytes/1D1a transduced T cells, n - indicated the number of mice with respective percent of Kb positive cells.

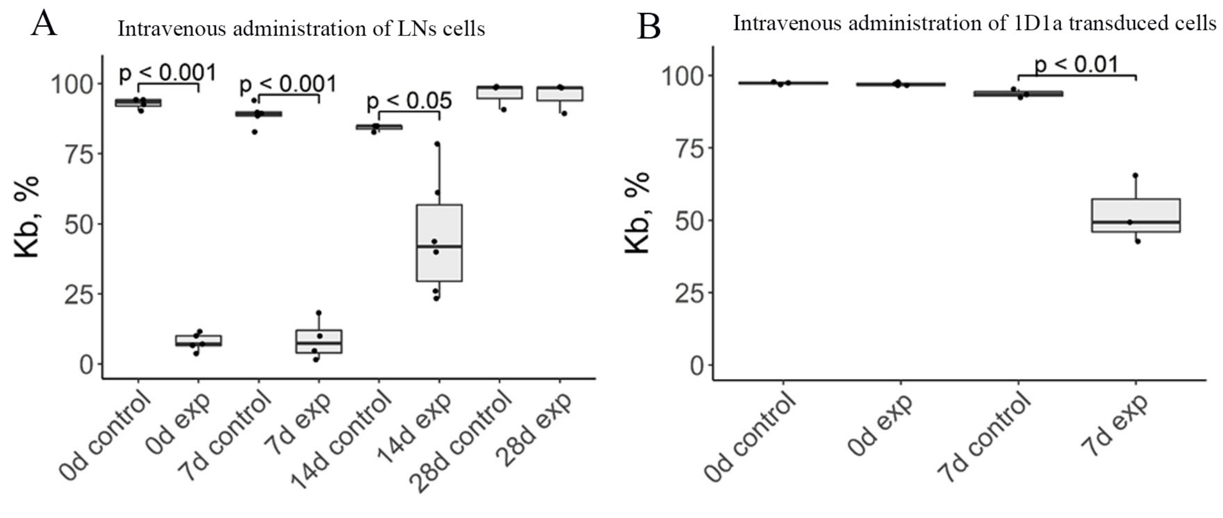

Supplementary Figure 6: Intravenous adoptive transfer of transgenic cells (**A**) and cells transduced with 1D1α (**B**). (**A**) 20 × 10^6 cells from LNs of WT and Tg mice were adoptively transferred into a vein of R101 mice. The immunization with EL-4 lymphoma cells was performed 0d, 7d, 14d, and 28d after the transfer. (**B**) Splenocytes of WT mice were transduced with 1D1α chain and separately with GFP to estimate the transduction efficiency. 5 × 10^6 transduced cells were adoptively transferred into a vein of R101 mice. The immunization with EL-4 lymphoma cells was performed 0d and 7d after the transfer. Analysis of the number of Kb positive cells in the peritoneal lavage was performed 6 days after immunization. Control - WT LNs/non-transduced T cells, exp (experiment) - Tg LNs/ 1D1a transduced T cells.
